# Automatic Seizure Detection Based on Time-Frequency Analysis and Artificial Neural Networks

**DOI:** 10.1155/2007/80510

**Published:** 2007-12-05

**Authors:** A. T. Tzallas, M. G. Tsipouras, D. I. Fotiadis

**Affiliations:** ^1^Department of Medical Physics, Medical School, University of Ioannina, GR 451 10 Ioannina, Greece; ^2^2Unit of Medical Technology and Intelligent Information Systems, Department of Computer Science, University of Ioannina, GR 451 10 Ioannina, Greece; ^3^Biomedical Research Institute, Foundation for Research and Technology-Hellas (BRI-FORTH), University of Ioannina, GR 451 10 Ioannina, Greece

## Abstract

The recording of seizures is of primary interest in the evaluation of epileptic patients. Seizure is the phenomenon of rhythmicity discharge from either a local area or the whole brain and the individual behavior usually lasts from seconds to minutes. Since seizures, in general, occur infrequently and unpredictably, automatic detection of seizures during long-term electroencephalograph (EEG) recordings is highly recommended. As EEG signals are nonstationary, the conventional methods of frequency analysis are not successful for diagnostic purposes. This paper presents a method of analysis of EEG signals, which is based on time-frequency analysis. Initially, selected segments of the EEG signals are analyzed using time-frequency methods and several features are extracted for each segment, representing the energy distribution in the time-frequency plane. Then, those features are used as an input in an artificial neural network (ANN), which provides the final classification of the EEG segments concerning the existence of seizures or not. We used a publicly available dataset in order to evaluate our method and the evaluation results are very promising indicating overall accuracy from 97.72% to 100%.

## 1. INTRODUCTION

Epilepsy is one of the most common neurological disorders with a prevalence of 0.6–0.8% of the world's population. Two-thirds of the patients achieve sufficient seizure control from anticonvulsive medication, and another 8–10% could benefit from resective surgery. For the remaining 25% of patients, no sufficient treatment is currently available [1]. The epilepsy is characterized by a sudden and recurrent malfunction of the brain, which is termed “seizure.” Epileptic seizures reflect the clinical signs of an excessive and hypersynchronous activity of neurons in the brain. Depending on the extent of the involvement of other brain areas during the course of the seizure, epilepsies can be divided into two main classes. Generalized seizures involve almost the entire brain, while focal (or partial) seizures originate from a circumscribed region of the brain (epileptic focus) and remain restricted to this region. Epileptic seizures may be accompanied by impairment or loss of consciousness: psychic, autonomic or sensory symptoms, or motor phenomena [2, 3].

Traditionally, suspected seizures are evaluated using a routine electroencephalogram (EEG), which is typically a 20-minute recording of the patient's brain waves. Because a routine EEG is of short duration, it is unlikely that actual events are recorded. Routine EEGs may record interictal hallmarks of epilepsy, including spikes, sharp waves, or spike-and-wave complexes. However, diagnostic difficulties arise when a person has a suspected seizure, or a neurological event of unclear etiology, not obvious in the routine EEG. The current gold standard is the continuous EEG recording along with video monitoring of the patient, which usually requires inpatient admission. This is a costly endeavour, which is not always available. The patient is away from his environment and routine, which may be associated with factors that provoke the patient's events [4]. The introduction of portable recording systems (ambulatory EEG), however, has allowed out-patient EEG recording to become more common. This has the advantage that patients are monitored in their normal environment without the reduction in seizure frequency usually occurring during in-patient sessions [4, 5].

Clinical neurophysiologists can then periodically review the EEG recordings and analyze the seizures that may have occurred during the monitoring session. However, reviewing a continuous EEG recording lasting several days can be a time-consuming process. In practice, the patient can indicate that a seizure occurs through the use of an alarm button, so that only the recording sections around the use of the button need to be analyzed. Unfortunately, in many cases, patients are not aware of the occurrence of their own seizures. An automated seizure detection system can thus be of great interest in identifying EEG sections that need to be reviewed. The main difficulty with it lies in the wide variety of EEG patterns that can characterize a seizure, such as “low-amplitude desynchronization, polyspike activity, rhythmic waves for a wide variety of frequencies and amplitudes, and spikes and waves” [6]. In extracranial recordings, EMG, movement, and eye blink artefacts often obscure seizures. Thus, from the pattern recognition point of view, the problem is extremely complex.

Research in automated seizure detection began in the 1970s and various algorithms addressing this problem [5–7] have been presented. Methods for automatic detection of seizures may rely on the identification of various patterns such as an increase in amplitude [8], sustained rhythmic activity [9, 10], or EEG flattening [11]. Several algorithms have been developed based on spectral [12–18] or wavelet features [19–23], amplitude relative to background activity [12, 24] and spatial context [24–27]. Chaotic features [28–31] such as correlation dimension [32, 33], Lyapunov exponents [34], and entropy [35] have also been proposed to characterize the EEG signal. These features can then be used to classify the EEG signal using statistical methods [28–30], nearest neighbour classifiers [36], decision trees [16], ANNs [21, 34], support vector machines (SVMs) [18, 37], or adaptive neurofuzzy inference systems [23, 35] in order to identify the occurrence of seizures. It is crucial for seizure detection systems to result in high sensitivity, even if this results in a large number of false detections. Such systems can then be used to reduce considerably the amount of data that need to be reviewed; neurophysiologists can then easily discard false detections.

In addition, to seizure detection systems, warning systems have also become increasingly valuable since detection of seizures at an early stage can warn the patient that a seizure is occurring. Also, they alert medical staff, and allow them to perform behavioral testing to further assess which specific functions may be impaired as a result of a seizure and help them in localizing the source of the seizure activity. Techniques used to forecast seizures include time-domain analysis [38], frequency-based methods [39], nonlinear dynamics and chaos [31, 40], methods of delays [41], and intelligent systems [42]. Advances in seizure prediction promise to give rise to implantable devices able to warn of impending seizures and to trigger therapy to prevent clinical epileptic attacks [2]. Treatments such as electrical stimulation of focal drug infusion could be given on demand and might eliminate side effects in some patients taking antiepileptic drugs.

Consequently, epileptic seizures give rise to changes in certain frequencies bands. Recent works have focused on the analysis of the **δ** (0.4–4 Hz), **θ** (4–8 Hz), **α** (8–12 Hz), **β** (12–30 Hz) rhythms, and their relation to epilepsy. An epileptic signal is nonstationary, having time-varying frequency components. Time-frequency (TF) representations combine both time and frequency information into a single representation and have proven to be powerful tools for the analysis of nonstationary signals [43], and have been used for neonatal seizure detection [44, 45].

In this work, we use TF analysis in order to extract several features from EEG segments, and subsequently use these features to classify the segments concerning epileptic seizures. The method is divided into three stages. Initially, TF analysis is performed for each EEG segment and its spectrum is acquired. Then, several features are extracted from it, measuring the fractional energy on specific TF windows. For this purpose, several partitions on the time axis and the frequency axis are tested. Finally, these features are used as inputs in an ANN, which provides the final classification according to the specified number of categories. A dataset of 500 EEG segments is used, while the method is evaluated for four different classification problems, each of them addressing a different interpretation of the medical problem and thus different selection of EEGs from the whole EEG segment dataset is required for each classification problem. TF analysis and feature extraction, reflecting the energy distribution over the TF plane, have been employed only for neonatal epileptic seizure detection and have not been previously applied in general epileptic seizure detection. In addition, no work addresses all four classification problems, which are directly related to the diagnosis provided by an expert. The obtained results indicate high accuracy compared to other existing approaches.

The rest of the paper is structured as follows. In Section 2, the dataset used in our work along with the employed methodology is described in detail. Then, the evaluation procedure and the obtained results are presented (Section 3), followed by an extensive discussion regarding them (Section 4). Finally, some concluding remarks are included in Section 5.

## 2. MATERIALS AND METHODS

**Figure 1:**
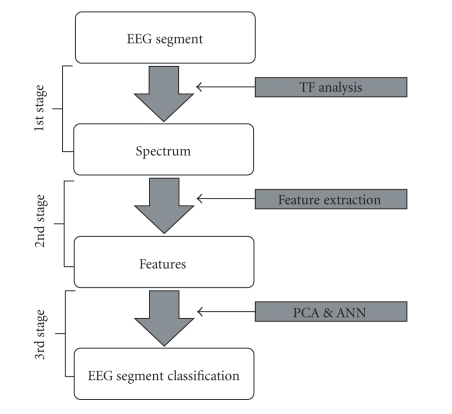
The flowchart of the proposed method.

The flowchart of the proposed method is shown in Figure 1. Below the dataset and its partitions used are briefly discussed and the three stages (time-frequency analysis, feature extraction, and classification) of the method are explained in detail.

### 2.1. Dataset

**Figure 2:**
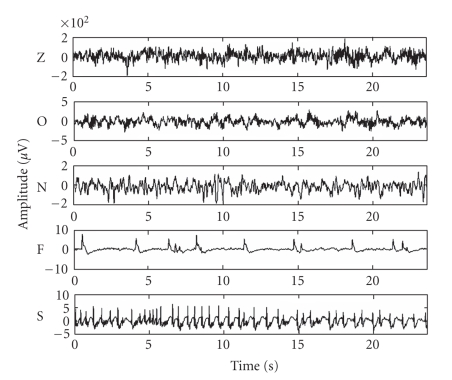
Exemplary EEG segments from each of the five subsets (Z, O, N, F, and S). From top to bottom: subset Z to subset S. The amplitudes of surface EEG recordings are typically in the order of some *μ*V. For intracranial EEG recordings, the amplitudes range around 100 *μ*V. For seizure activity, these voltages can exceed 1000 *μ*V.

An EEG dataset, which is available online [46] and includes recordings for both healthy and epileptic subjects, is used. The dataset includes five subsets (denoted as Z, O, N, F, and S) each containing 100 single-channel EEG segments, each one having 23.6-second duration. The subsets Z and O have been acquired using surface EEG recordings of five healthy volunteers with eyes open and closed, respectively. Signals in two sets have been measured in seizure-free intervals from five patients in the epileptogenic zone (set F) and from the hippocampal formation of the opposite hemisphere of the brain (set N). Finally, subset S contains seizure activity, selected from all recording sites exhibiting ictal activity. Subsets Z and O have been recorded extracranially, using standard electrode positioning (according to the international 10–20 system [47]), whereas subsets N, F, and S have been recorded intracranially. More specifically, depth electrodes are implanted symmetrically into the hippocampal formation. EEG segments of subsets N and F were taken from all contacts of the relevant depth electrode [46]. In addition, strip electrodes are implanted onto the lateral and basal regions (middle and bottom) of the neocortex. EEG segments of the subsets S were taken from contacts of all electrodes (depth and strip). All EEG signals were recorded with the same 128-channel amplifier system, using an average common reference. The data were digitized at 173.61 samples per second using 12 bit resolution and they have the spectral bandwidth of the acquisition system, which varies from 0.5 Hz to 85 Hz. Typical EEG segments (one from each category of the dataset) are shown in Figure 2.

In our analysis, we use the above-described dataset to create four different classification problems and then we tested our method with all of them.

1. In the first, all the EEG segments from the dataset were used and they were classified into three different classes: Z and O types of EEG segments were combined to a single class, N and F types were also combined to a single class, and type S was the third class. This set is the one closest to real medical applications including three categories; normal (i.e., types Z and O), seizure-free (i.e., types N and F) and seizure (i.e., type S).
2. In the second, again all the EEG segments from the dataset were used and they were classified into two different classes: Z, O, N, and F types are included in the first class and type S in the second class. This is also close to real medical applications, being slightly simpler than the previous, classifying the EEG segments into nonseizures and seizures.
3. The third has similar classes with the first, that is, normal, seizure-free and seizure, but not all the EEG segments from the dataset were employed. The normal class includes only the Z-type EEG segments, the seizure-free class the F-type EEG segments, and the seizure class the S-type.
4. The fourth has similar classes with the second, that is, normal and seizure, but again not all the EEG segments from the dataset were employed. The normal class includes only the Z-type EEG segments while the seizure class includes the S-type.

The above classification problems are shown in detail in Table 1.

### 2.2. Time-frequency analysis

**Figure 3:**
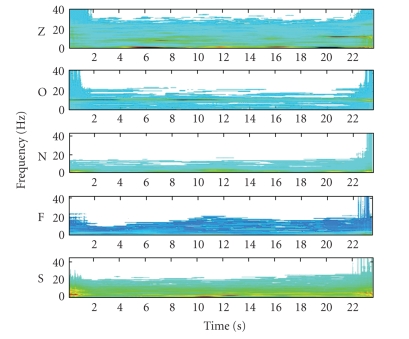
The obtained spectrum for five EEG segments, one for each of the original dataset categories (Z, O, N, F, and S).

In the proposed method, the smoothed pseudo-Wigner-Ville distribution (SPWVD) [48, 49] is applied to each EEG segment, defined as

(1) $$\begin{array}{ll} & \text{SPWV}{\text{D}}_{x}\left(t,\omega \right)\\ & =\int_{-\infty }^{+\infty }h\left(\tau \right)\left(\int_{-\infty }^{+\infty }g\left(s-t\right)x\left(s+\frac{\tau }{2}\right)x^{*}\left(s-\frac{\tau }{2}\right)ds\right)e^{-j2\pi \omega \tau }d\tau , \end{array}$$

where x(⋅) is the signal, t is the time, ω is the frequency, and g(⋅) and h(⋅) are time and frequency smoothing window functions, respectively. SPWVD can substantially suppress the cross terms, which is a major limitation of the time-frequency analysis. The time smoothing window was selected to be a Hamming 64-point length window, which was the same for all tests performed for evaluation. The length of the frequency smoothing window is not always the same; we have selected several different frequency resolutions (64, 128, 256, and 512 points length window), and we tested the method for all of them. Time-frequency (TF) analysis is used to calculate the spectrum of the signal. Figure 3 shows the spectrum of five EEG segments, one of each of the original dataset categories (Z, O, N, F, and S), using a 512-point length window.

### 2.3. Feature extraction

**Figure 4:**
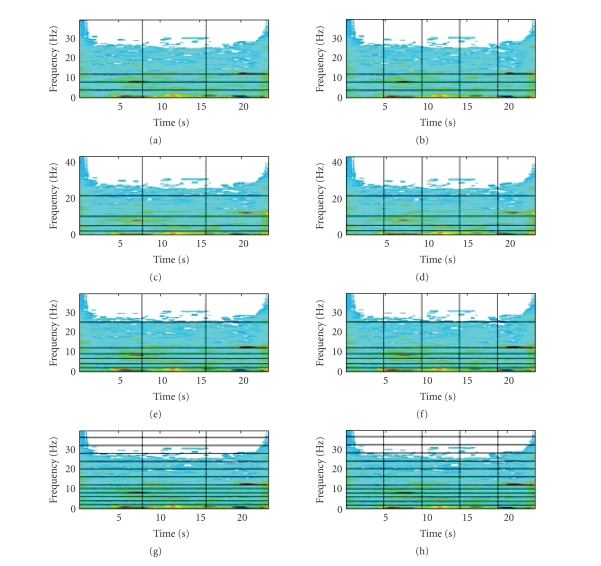
The spectrums obtained for various combinations of time and frequency partitions: (a) 3 time windows and 4 frequency subbands, (b) 5 time windows and 4 frequency subbands, (c) 3 time windows and 5 frequency subbands, (d) 5 time windows and 5 frequency subbands, (e) 3 time windows and 7 frequency subbands, (f) 5 time windows and 7 frequency subbands, (g) 3 time windows and 13 frequency subbands, and (h) 5 time windows and 13 frequency subbands.

The spectrum of the signals, computed using TF analysis, is used to extract several features. To do that, a grid is used, based on a time and a frequency partition. In the time domain, two different partitions were used, having three and five equal-sized windows, respectively, while in the frequency domain, four different partitions were used, which divide the frequency domain in 4, 5, 7, and 13 subbands. These subbands, which are not always equal, are shown in Table 2 and they are created using medical knowledge about the EEG and the features that are expected to be found in certain frequency bands for the specific types of EEG segments included in the original dataset. All the combinations between these time and frequency partitions are used, in order to extract several sets of features. The result of the application of TF analysis in an EEG segment for different combinations of time windows and frequency subbands is shown in Figure 4.

Each feature, f(i,j), is calculated as

(2) $$f\left(i,j\right)=\int_{t_{i}}\int_{\omega_{j}}\text{SPWV}{\text{D}}_{x}\left(t,\omega \right)d\omega \;dt,$$

where $t_{i}$ is the *i*th time window and $\omega_{j}$ is the *j*th frequency band. Each feature represents the fractional energy of the signal in a specific frequency band and time window; thus the total feature set depicts the distribution of the signal's energy over the TF plane. Therefore, it is expected that each feature set carries sufficient information related to the nonstationary properties of the signal and thus, it can be useful for the classification process. The feature set initially is represented as an N×M matrix, where N is the number of time windows and M is the number of frequency subbands, and then it is reshaped into an N⋅M size vector. The length of the feature vector is not the same in all cases and it depends only on the time and frequency partitions. In all cases, an additional feature is used, which is the total energy of the signal. Thus, in each case the total number of features is N⋅M+1.

### 2.4. Classification

The calculated features are fed into a feed-forward artificial neural network (ANN). To reduce the dimensionality of the input patterns, principal component analysis (PCA) is employed with the threshold set to 1%. The architecture of the neural network is different in each classification problem: N inputs (N is the number of features resulted from the PCA), one hidden layer with 4*N neurons, and M outputs (M is the number of the classes), each of them being a real number in the interval [0, 1]. The units in the hidden layer are sigmoid units with hyperbolic tangent as activation function, while the outputs are linear. Half of the patterns of the dataset were randomly selected to be used for training, while the rest were used for testing. The network is trained using a standard backpropagation algorithm [50]. Ten different training-test sets were created for each classification problem and thus ten different neural networks were optimized. The final result is obtained as the average of their results.

## 3. RESULTS

The four classification problems, described above, are used to evaluate the proposed method. For each of them, all combinations between frequency resolutions (64, 128, 256, or 512), time windows (3 or 5), and frequency bands (4, 5, 7, or 13) were tested; totally 32 different combinations for each classification problem. For each problem, half of the EEG segments, randomly selected, were used for the training of the neural network, while the other half for testing.

The size of the confusion matrix depends on the classification problem: 3 × 3 for problems (1) and (3), 2 × 2 for problems (2) and (4). Results for each class *i* are derived in terms of sensitivity (Sens), specificity (Spec), and selectivity (Sel):

(3) $$\begin{array}{ll} & \text{Sen}{\text{s}}_{i}\\ & =\frac{\text{Number}\;\text{of}\;\text{patterns }\text{of}\;\text{class}\;i\;\text{classified}\;\;\text{in}\;\text{class}\;\;i}{\text{Total}\;\text{number}\;\text{of}\;\text{patterns}\;\;\text{in}\;\text{class}\;\;i\;}, \end{array}$$

(4) $$\begin{array}{ll} & \text{Spe}{\text{c}}_{i}\\ & =\frac{\text{Number}\;\text{of}\;\text{patterns}\;\;\text{not}\;\text{in}\;\text{class}\;\;i\;\text{classified}\;\;\;\text{not}\;\text{in}\;\text{class}\;\;i}{\text{Total}\;\text{number}\;\text{of}\;\text{patterns}\;\;\text{not}\;\text{in}\;\text{class}\;\;i\;}, \end{array}$$

(5) $$\begin{array}{ll} & \text{Se}{\text{l}}_{i}\\ & =\frac{\text{Number}\;\text{of}\;\text{patterns}\;\;\text{of}\;\text{class}\;\;i\;\text{classified}\;\;\text{in}\;\text{class}\;\;i}{\text{Total}\;\text{number}\;\text{of}\;\text{patterns}\;\;\text{classified}\;\;\text{in}\;\text{class}\;\;i\;}. \end{array}$$

The results for the classification problems (1)–(4) are shown in Tables 3–6, respectively.

The accuracy (Acc), defined as

(6) Acc=Trace(cm),

where cm is the confusion matrix, defined as

(7) $$\begin{array}{ll} \text{c}{\text{m}}_{i,j} & =\text{number}\;\text{of}\;\text{patterns}\;\text{belonging}\;\text{to}\;\text{class}\;i\\ & \text{ and}\;\text{classified}\;\text{to}\;\text{class}\;j, \end{array}$$

is calculated for each confusion matrix. The computed accuracies, along with the standard deviations are presented in Table 7. Additionally, the initial number of features and the reduced number of features after the PCA application are presented. For each classification problem, overall results have been derived, that is, the maximum and minimum accuracies (for all combinations between frequency resolutions, time windows, and frequency subbands) as well as the average accuracy and the standard deviation. For the first classification problem, the best obtained accuracy is 97.72%, achieved for 512 frequency resolution, 3 time windows, and 13 frequency subbands. For the second classification problem, the best obtained accuracy is 97.73%, achieved for 512 frequency resolution, 3 time windows, and 5 frequency subbands. For the third classification problem, the best obtained accuracy is 99.28%, achieved for 128 frequency resolution, 3 time windows, and 4 frequency subbands. Finally, for the fourth classification problem, the best obtained accuracy is 100%, achieved in most of the cases; in 28 out of 32 different evaluations of the fourth classification problem we obtained accuracy 100%.

For the first two classification problems, the obtained accuracies of the different evaluations varied significantly; almost 6.5% (max-min) for both of them, with average 95% and standard deviation 1.7%. For the third classification problem, the max-min difference is 3% and the average 97.94%, with 0.75% standard deviation. Finally, for the fourth classification problem, the max-min difference is 1.3% and the average 99.92%, with 0.26% standard deviation.

## 4. DISCUSSION

We have proposed an automated method for seizure detection in EEG recordings. The method is based on TF analysis of the EEG segments and extraction of several features from the spectrum of the signal. These features are fed into neural networks, which provide the final classification of the EEG segments. The method is evaluated using four different classification problems originated from the type of medical diagnosis, which can be obtained. The effect of different parameters of the method on the classification accuracy is examined. Those parameters are the frequency resolution of the TF analysis, the length of the time window, and the width of the frequency subbands used in the feature extraction. The different combinations among all the afore-mentioned parameters result in a large number of different experimental settings (32) for each classification problem (4) and 10 different realizations (selections of training/test datasets) for each of them—totally 1280 optimized and evaluated ANNs—and results are presented for all of them. This is considered an extensive validation procedure, which can sufficiently exploit the potentials of the proposed method.

In this method, the SPWVD has been employed for the TF analysis of the EEG signals. Other distributions have been also tried but the better results were obtained for SPWVD.

The frequency resolution, used in the TF analysis, does not greatly affect the accuracy of the proposed method; the average accuracies of all different combinations of time windows and frequency subbands, for the four classification problems, are 96.71%, 97.13%, 96.7%, and 96.87% for 64, 128, 256, and 512 points length windows, respectively. It is obvious that the use of 128 points length window slightly improves the results. On the other hand, the number of the time windows is important for the analysis; in the case of three time windows, the average accuracy of all different combinations between the frequency resolutions and frequency subbands, for all four classification problems, is 97.52%, while the accuracy in the case of five time windows is 96.2%. This means that analyzing EEG segments of approximately 8-second length reveals more information for the epileptic seizures than having 5-second windows. Other statistical measurements lead to the same conclusion; in the case of three time windows, the minimum accuracy of all cases is 93.04% and the standard deviation 1.8%, while the accuracy for five time windows is 91.08% and the standard deviation 2.9%, respectively. Finally, concerning the number of frequency subbands, again the reported average accuracies for all combinations among the frequency resolutions and the time windows, for all classification problems, are 97.07%, 96.87%, 96.84%, and 96.62% for 4, 5, 7, and 13 frequency subbands, respectively. This gives indications that the separation in δ, θ, α, and β rhythms is the one that mostly detects the TF components that characterize the signal regarding epileptic seizures, compared to 5 and 7, which have been used in other methods [20, 22], and 13, which is defined in this work to examine if a frequency resolution with a large number of frequency subbands improves the classification accuracy. The results indicate that all selections for frequency subbands result in similar high-average accuracies—the difference between the best and worst age accuracy is 0.45%. This can be justified since they are generated either based on expert knowledge or have been previously proposed in the literature. Concerning the frequency subbands, the higher their number, is the lower (slightly) the average accuracy obtained.

To our knowledge, TF analysis and feature extraction, which reflect the energy over the TF plane, have been only applied in the analysis of neonatal EEG signals (and mainly for neonatal epileptic seizure detection) and not EEG signals in general. Moreover, the quality of the proposed method can be proved from the obtained results. The accuracy achieved by our method for the epileptic seizure detection is more than satisfactory and also its automated nature makes it suitable to be used in real clinical conditions. Besides the feasibility of a real-time implementation of the proposed method, the diagnosis can be made more accurate by increasing the number of parameters. A system that may be developed as a result of this study may provide feedback to the experts for classification of the EEG signals quickly and accurately by examining the EEG signal.

Table 8 presents a comparison between our method and other methods proposed in the literature. Only methods evaluated in the same dataset are included so that a comparison between the results is feasible. For the two classes' problem, using only the Z and S types of EEG segments, the results obtained from the evaluation of our method are the best presented for this dataset. The difference between our result and all other results proposed in the literature varies from 0.4% to 10%. The second two classes' problem that we used to evaluate our method also presents high-accuracy results (97.73%). It is worth to mention here that a method that discriminates EEGs into nonseizure and seizure is much closer to the expert needs.

Regarding the three classes' problem, the results obtained from our method are the best presented for this dataset, either using only the Z, F, and S types or all the available dataset. In the case of using the third problem to evaluate our method (i.e., only the Z, F, and S types), the difference between our results and all others' results varies from 2.5% to 13.4%. In the case of using the first classification problem to evaluate our method (i.e., the Z and O, F and N, S types), the difference between our results and all others' results ranges from 1% to 12%. The second case has also the advantage of being a more realistic classification, dividing the dataset to normal, seizure-free, and seizure EEGs, and thus being closer to clinical conditions.

Still, however, there are several other aspects either technical or medical which must be addressed. From the technical point of view, although we have examined the effect of various parameters (frequency resolution, number of time windows, and frequency bands), some other, like time-frequency distributions (e.g., reduced interference distributions), have not been explored. Furthermore, we mainly focused on the effects of the parameters related to frequency analysis, either for the calculation of the spectrum of the signal or for the frequency resolution for feature extraction. More detailed examination of the time resolution for feature extraction may also reveal important information regarding the seizure detection; this feature will be addressed in feature communications. From the medical point of view, the most important feature is that currently the method is used to characterize predetermined (with respect to their length) EEG segments. An important aspect is also the modification of the proposed method in order to be able to automatically detect highly suspicious segments (regardless of their length) into long time EEG recordings and classify them.

## 5. CONCLUSIONS

In this paper, we explored the ability of the TF analysis to classify EEG segments which contain epileptic seizures. We have extracted several time-frequency features and we examined the effect of the parameters entering the problem, that is, the frequency resolution of the time-frequency analysis and the number of time windows and frequency subbands used for feature extraction. Promising results have been reported after the evaluation of the proposed method in four different classification problems, derived from a well-known database. However, several types of artefacts have been removed from this database after visual inspection. This is a limitation of the evaluation of our method and thus further evaluation under real clinical conditions is required in order to fully exploit its potential. Another limitation is that in the current study high-frequency components (over 40 Hz) were not measured and thus taken under consideration; the employment of high-frequency components, such as gamma activity, and their importance concerning epileptic seizure detection will be addressed in a future communication. Finally, several technical aspects can be further investigated, such as different techniques for feature reduction and alternative classification algorithms.

## Figures and Tables

**Table 1:** The classes and the corresponding number of EEG segments of the four classification problems.

Classification problem	Classes	Number of EEG segments
1	Normal (Z, O)	200
	Seizure-free (N, F)	200
	Seizure (S)	100

	Total	500

2	Nonseizure (Z, O, N, F)	400
	Seizure (S)	100

	Total	500

3	Normal (Z)	100
	Seizure-free (N)	100
	Seizure (S)	100

	Total	300

4	Normal (Z)	100
	Seizure (S)	100

	Total	200

**Table 2:** The frequency ranges (Hz) of four frequency subbands (4, 5, 7, and 13).

	Frequency subbands			
	4	5	7	13
Frequency ranges (Hz)	0–4	0–2.5	0–2	0–2
	4–8	2.5–5.5	2–4	2–4
	8–12	5.5–10.5	4–6.5	4–6
	12–40	10.5–21.5	6.5–9	6–8
	—	21.5–43.5	9–12	8–10
	—	—	12–25	10–12
	—	—	25–40	12–16
	—	—	—	16–20
	—	—	—	20–24
	—	—	—	24–28
	—	—	—	28–32
	—	—	—	32–36
	—	—	—	36–40

**Table 3:** Results for the first classification problem, in terms of sensitivity (Sens), specificity (Spec), and selectivity (Sel) in % values. Those are given for all TF resolutions (64, 128, 256, and 512), time windows (3 and 5), and frequency subbands (4, 5, 7, and 13).

Frequency subbands					4			5			7			13		
Classes					ZO	NF	S	ZO	NF	S	ZO	NF	S	ZO	NF	S
Frequency resolution	64	Time windows	3	Sens	98.90	94.20	97.00	97.90	96.80	95.60	95.80	93.20	93.80	96.10	97.80	90.80
				Spec	98.00	98.53	98.40	97.53	97.80	99.75	95.67	95.80	99.35	98.27	95.53	99.30
				Sel	97.06	97.72	93.81	96.36	96.70	98.96	93.65	93.67	97.30	97.37	93.59	97.01
			5	Sens	95.40	91.90	90.80	95.70	95.50	88.80	96.20	92.90	92.20	93.30	93.30	88.60
				Spec	95.53	95.07	98.40	96.20	94.40	99.85	96.27	96.27	98.20	95.33	93.87	98.55
				Sel	93.44	92.55	93.42	94.38	91.92	99.33	94.50	94.31	92.76	93.02	91.02	93.86

Frequency resolution	128	Time windows	3	Sens	99.20	95.50	97.40	97.90	95.20	91.20	99.60	96.90	94.60	96.80	93.20	87.40
				Spec	97.60	98.87	99.35	96.80	96.80	99.15	98.00	98.13	99.80	95.47	95.47	98.65
				Sel	96.50	98.25	97.40	95.33	95.20	96.41	97.08	97.19	99.16	93.44	93.20	94.18
			5	Sens	95.90	92.70	97.40	96.80	93.10	92.00	96.30	93.60	89.80	96.20	91.60	93.00
				Spec	96.73	96.47	98.75	95.67	96.40	98.90	95.33	96.07	98.85	94.93	97.60	97.75
				Sel	95.14	94.59	95.12	93.71	94.52	95.44	93.22	94.07	95.13	92.68	96.22	91.18

Frequency resolution	256	Time windows	3	Sens	96.60	95.70	98.00	98.20	90.80	87.20	98.00	96.00	96.20	96.50	98.00	93.00
				Spec	98.27	97.20	99.05	93.53	95.87	99.25	97.20	97.93	99.70	97.60	97.67	99.05
				Sel	97.38	95.80	96.27	91.01	93.61	96.67	95.89	96.87	98.77	96.40	96.55	96.07
			5	Sens	95.20	93.50	93.80	94.90	92.40	89.80	94.00	91.90	92.80	96.80	92.00	85.00
				Spec	96.80	95.40	98.65	95.27	94.13	99.05	95.53	94.73	98.45	92.47	97.33	98.30
				Sel	95.20	93.13	94.56	93.04	91.30	95.94	93.35	92.08	93.74	89.55	95.83	92.59

Frequency resolution	512	Time windows	3	Sens	97.50	95.00	93.80	98.50	97.30	91.60	97.30	95.70	96.40	**98.80**	**99.00**	**93.00**
				Spec	98.20	96.67	98.55	97.33	98.13	99.20	98.53	97.20	98.80	**98.20**	**98.20**	**99.85**
				Sel	97.31	95.00	94.18	96.10	97.20	96.62	97.79	95.80	95.26	**97.34**	**97.35**	**99.36**
			5	Sens	95.70	90.20	95.80	95.60	92.70	90.00	92.30	90.40	90.00	96.00	95.70	83.20
				Spec	95.47	96.27	98.10	95.27	95.93	98.25	93.60	93.27	98.70	95.67	94.13	99.30
				Sel	93.37	94.15	92.65	93.09	93.83	92.78	90.58	89.95	94.54	93.66	91.58	96.74

**Table 4:** Results for the second classification problem, in terms of sensitivity (Sens), specificity (Spec), and selectivity (Sel) in % values. Those are given for all TF resolutions (64, 128, 256, and 512), time windows (3 and 5), and frequency subbands (4, 5, 7, and 13).

Frequency subbands					4		5		7		13	
Classes					ZONF	S	ZONF	S	ZONF	S	ZONF	S
Frequency resolution	64	Time windows	3	Sens	98.40	97.60	98.55	95.60	99.30	96.00	99.10	92.40
				Spec	97.60	98.40	95.60	98.55	96.00	99.30	92.40	99.10
				Sel	99.39	93.85	98.90	94.28	99.00	97.17	98.12	96.25
			5	Sens	97.70	97.00	99.35	93.20	98.70	91.80	98.65	91.80
				Spec	97.00	97.70	93.20	99.35	91.80	98.70	91.80	98.65
				Sel	99.24	91.34	98.32	97.29	97.97	94.64	97.96	94.44

Frequency resolution	128	Time windows	3	Sens	99.50	98.40	99.25	92.60	99.55	96.80	98.05	92.40
				Spec	98.40	99.50	92.60	99.25	96.80	99.55	92.40	98.05
				Sel	99.60	98.01	98.17	96.86	99.20	98.17	98.10	92.22
			5	Sens	99.50	97.80	99.05	92.80	98.95	93.20	98.10	94.80
				Spec	97.80	99.50	92.80	99.05	93.20	98.95	94.80	98.10
				Sel	99.45	98.00	98.22	96.07	98.31	95.69	98.69	92.58

Frequency resolution	256	Time windows	3	Sens	99.25	99.00	98.90	86.40	99.45	96.60	99.40	93.60
				Spec	99.00	99.25	86.40	98.90	96.60	99.45	93.60	99.40
				Sel	99.75	97.06	96.68	95.15	99.15	97.77	98.42	97.50
			5	Sens	98.55	96.20	99.00	94.40	98.70	94.20	97.15	92.60
				Spec	96.20	98.55	94.40	99.00	94.20	98.70	92.60	97.15
				Sel	99.05	94.31	98.61	95.93	98.55	94.77	98.13	89.04

Frequency resolution	512	Time windows	3	Sens	98.90	96.20	**99.05**	**94.20**	98.85	95.60	99.70	94.20
				Spec	96.20	98.90	**94.20**	**99.05**	95.60	98.85	94.20	99.70
				Sel	99.05	95.63	**98.56**	**96.12**	98.90	95.41	98.57	98.74
			5	Sens	98.35	95.00	98.65	92.60	98.75	93.40	98.85	89.20
				Spec	95.00	98.35	92.60	98.65	93.40	98.75	89.20	98.85
				Sel	98.74	93.50	98.16	94.49	98.36	94.92	97.34	95.10

**Table 5:** Results for the third classification problem, in terms of sensitivity (Sens), specificity (Spec), and selectivity (Sel) in % values. Those are given for all TF resolutions (64, 128, 256, and 512), time windows (3 and 5), and frequency subbands (4, 5, 7, and 13).

Frequency subbands					4			5			7			13		
Classes					Z	F	S	Z	F	S	Z	F	S	Z	F	S
Frequency resolution	64	Time windows	3	Sens	99.00	93.80	96.60	97.80	95.20	98.20	97.00	87.80	97.80	97.40	98.00	93.20
				Spec	98.90	97.80	98.00	98.60	98.50	98.50	94.60	98.30	98.40	99.30	94.60	98.90
				Sel	97.83	95.52	96.02	97.22	96.95	97.04	89.98	96.27	96.83	98.54	90.07	97.69
			5	Sens	94.60	84.20	96.40	96.40	92.40	92.80	95.20	92.80	94.80	90.20	90.60	93.60
				Spec	94.50	96.20	96.90	95.80	95.30	99.70	97.40	95.50	98.50	96.40	92.80	98.00
				Sel	89.58	91.72	93.96	91.98	90.77	99.36	94.82	91.16	96.93	92.61	86.29	95.90

Frequency resolution	128	Time windows	3	Sens	**99.20**	**90.60**	**97.40**	99.40	93.40	92.80	99.40	98.60	93.00	97.40	95.80	93.40
				Spec	**96.50**	**98.50**	**98.60**	97.00	97.50	98.30	98.60	97.50	99.40	98.50	96.40	98.40
				Sel	**93.41**	**96.79**	**97.21**	94.31	94.92	96.47	97.26	95.17	98.73	97.01	93.01	96.69
			5	Sens	95.40	91.60	95.60	98.20	96.20	92.20	95.00	95.40	95.20	96.40	90.60	94.00
				Spec	97.30	95.60	98.40	97.80	96.00	99.50	98.30	96.00	98.50	95.40	97.80	97.30
				Sel	94.64	91.24	96.76	95.71	92.32	98.93	96.54	92.26	96.95	91.29	95.37	94.57

Frequency resolution	256	Time windows	3	Sens	92.00	92.80	98.80	99.20	96.20	92.60	96.40	96.00	94.00	98.20	97.80	95.20
				Spec	97.90	95.40	98.50	97.90	96.50	99.60	97.80	96.60	98.80	98.40	97.80	99.40
				Sel	95.63	90.98	97.05	95.94	93.22	99.14	95.63	93.39	97.51	96.84	95.69	98.76
			5	Sens	95.60	91.40	95.40	90.20	92.80	94.40	94.20	91.00	92.40	98.40	91.80	97.00
				Spec	97.40	95.70	98.10	97.10	93.00	98.60	95.70	94.50	98.60	96.60	98.20	98.80
				Sel	94.84	91.40	96.17	93.96	86.89	97.12	91.63	89.22	97.06	93.54	96.23	97.59

Frequency resolution	512	Time windows	3	Sens	99.80	95.20	96.20	99.60	97.40	96.20	94.20	94.20	94.60	99.40	96.40	93.80
				Spec	98.20	98.30	99.10	98.40	98.60	99.60	98.00	95.20	98.30	98.30	97.30	99.20
				Sel	96.52	96.55	98.16	96.89	97.21	99.18	95.93	90.75	96.53	96.69	94.70	98.32
			5	Sens	97.60	85.80	95.40	98.00	91.20	94.00	91.40	92.60	96.40	96.20	94.00	89.40
				Spec	94.70	96.60	98.10	97.50	96.50	97.60	97.90	94.50	97.80	96.50	94.20	99.10
				Sel	90.20	92.66	96.17	95.15	92.87	95.14	95.61	89.38	95.63	93.22	89.02	98.03

**Table 6:** Results for the fourth classification problem, in terms of sensitivity (Sens), specificity (Spec), and selectivity (Sel) in % values. Those are given for all TF resolutions (64, 128, 256, and 512), time windows (3 and 5), and frequency subbands (4, 5, 7, and 13).

Frequency subbands					4		5		7		13	
Classes					Z	S	Z	S	Z	S	Z	S
Frequency resolution	64	Time windows	3	Sens	**100**	**100**	**100**	**100**	**100**	**100**	**100**	**100**
				Spec	**100**	**100**	**100**	**100**	**100**	**100**	**100**	**100**
				Sel	**100**	**100**	**100**	**100**	**100**	**100**	**100**	**100**
			5	Sens	**100**	**100**	**100**	**100**	**100**	**100**	**100**	**100**
				Spec	**100**	**100**	**100**	**100**	**100**	**100**	**100**	**100**
				Sel	**100**	**100**	**100**	**100**	**100**	**100**	**100**	**100**

Frequency resolution	128	Time windows	3	Sens	**100**	**100**	**100**	**100**	**100**	**100**	100	99.80
				Spec	**100**	**100**	**100**	**100**	**100**	**100**	99.80	100
				Sel	**100**	**100**	**100**	**100**	**100**	**100**	99.80	100
			5	Sens	**100**	**100**	**100**	**100**	**100**	**100**	**100**	**100**
				Spec	**100**	**100**	**100**	**100**	**100**	**100**	**100**	**100**
				Sel	**100**	**100**	**100**	**100**	**100**	**100**	**100**	**100**

Frequency resolution	256	Time windows	3	Sens	**100**	**100**	99.80	97.60	**100**	**100**	100	98.80
				Spec	**100**	**100**	97.60	99.80	**100**	**100**	98.80	100
				Sel	**100**	**100**	97.65	99.80	**100**	**100**	98.81	100
			5	Sens	**100**	**100**	**100**	**100**	**100**	**100**	**100**	**100**
				Spec	**100**	**100**	**100**	**100**	**100**	**100**	**100**	**100**
				Sel	**100**	**100**	**100**	**100**	**100**	**100**	**100**	**100**

Frequency resolution	512	Time windows	3	Sens	**100**	**100**	**100**	**100**	**100**	**100**	100	99.00
				Spec	**100**	**100**	**100**	**100**	**100**	**100**	99.00	100
				Sel	**100**	**100**	**100**	**100**	**100**	**100**	99.01	100
			5	Sens	**100**	**100**	**100**	**100**	**100**	**100**	**100**	**100**
				Spec	**100**	**100**	**100**	**100**	**100**	**100**	**100**	**100**
				Sel	**100**	**100**	**100**	**100**	**100**	**100**	**100**	**100**

**Table 7:** Accuracy (%), standard deviation (in the parenthesis), and initial number of features/reduced number of features after PCA application, for all classification problems (1, 2, 3, and 4) reported, for all TF resolutions (64, 128, 256, and 512), time windows (3 and 5), and frequency subbands (4, 5, 7, and 13).

Frequency resolution	Time windows	Frequency subbands	Classification problem			
			1	2	3	4
64	3	4	96.64 (0.34) 13/3	96.47(0.45) 13/3	98.24 (0.33) 13/3	**100** **(0) 13/3**
		5	97 (0.76) 16/3	97.07(0.78) 16/3	97.96 (0.61) 16/3	**100** **(0) 16/3**
		7	94.36 (0.58) 22/5	94.2 (0.89) 22/5	98.64 (0.34) 22/5	**100** **(0) 22/5**
		13	95.72 (0.71) 40/4	95.2 (1.25) 40/4	97.76 (0.33) 40/4	**100** **(0) 40/4**
	5	4	93.08 (0.96) 21/4	91.73 (0.84) 21/4	97.56 (0.39) 21/4	**100** **(0)** **21/4**
		5	94.24 (0.54) 26/4	93.87 (1.08) 26/4	98.12 (0.6) 26/4	**100** **(0) 26/4**
		7	94.08 (0.7) 36/4	94.27 (0.95) 36/4	97.32 (0.19) 36/4	**100** **(0) 36/4**
		13	92.36 (0.81) 66/4	91.47 (0.82) 66/4	97.28 (0.37) 66/4	**100** **(0) 66/4**

128	3	4	97.36 (0.34) 13/3	95.73 (0.47) 13/3	**99.28** **(0.17)** **13/3**	**100** **(0) 13/3**
		5	95.48 (0.33) 16/3	95.2 (0.61) 16/3	97.92 (0.32) 16/3	**100** **(0)** **16/3**
		7	97.52 (0.25) 22/4	97 (0.47) 22/4	99 (0.34) 22/4	**100** **(0) 22/4**
		13	93.48 (0.80) 40/5	95.53 (1.3) 40/5	96.92 (0.42) 40/5	99.9 (0.32) 40/5
	5	4	94.92 (0.71) 21/4	94.2 (1.41) 21/4	99.16 (0.35) 21/4	**100** **(0)** 21/4
		5	94.36 (0.72) 26/4	95.53 (0.71) 26/4	97.8 (0.28) 26/4	**100** **(0) 26/4**
		7	93.92 (1.1) 36/4	95.2 (0.93) 36/4	97.8 (0.39) 36/4	**100** **(0) 36/4**
		13	93.72 (0.9) 66/5	93.67 (1.18) 66/5	97.44 (0.47) 66/5	**100** **(0) 66/5**

256	3	4	96.52 (0.27) 13/3	94.53 (0.42) 13/3	99.2 (0) 13/3	**100** **(0) 13/3**
		5	93.04 (0.78) 16/3	96 (0.7) 16/3	96.4 (0.53) 16/3	98.7 (0.82) 16/3
		7	96.84 (0.35) 22/5	95.47 (0.53) 22/5	98.88 (0.41) 22/5	**100** **(0) 22/5**
		13	96.4 (0.9) 40/6	97.07 (0.84) 40/6	98.24 (0.39) 40/6	99.4 (0.52) 40/6
	5	4	94.24 (0.8) 21/4	94.13 (1.21) 21/4	98.08 (0.53) 21/4	**100** **(0)** **21/4**
		5	92.88 (0.53) 26/5	92.47 (1.18) 26/5	98.08 (0.49) 26/5	**100** **(0)** **26/5**
		7	92.92 (0.6) 36/5	92.53 (0.61) 36/5	97.8 (0.43) 36/5	**100** **(0) 36/5**
		13	92.52 (0.71) 66/5	95.73 (0.84) 66/5	96.24 (0.63) 66/5	**100** **(0) 66/5**

512	3	4	95.76 (0.28) 13/3	97.07 (0.72) 13/3	98.36 (0.4) 13/3	**100** **(0) 13/3**
		5	96.64 (0.34) 16/4	**97.73** **(1) 16/4**	98.08 (0.62) 16/4	**100** **(0) 16/4**
		7	96.48 (0.59) 22/5	94.33 (0.85) 22/5	98.2 (0.28) 22/5	**100** **(0) 22/5**
		13	**97.72** **(0.38) 40/6**	96.53 (0.69) 40/6	98.6 (0.47) 40/6	99.5 (0.53) 40/6
	5	4	93.52 (0.67) 21/5	92.93 (0.9) 21/5	97.68 (0.41) 21/5	**100** **(0) 21/5**
		5	93.32 (0.46) 26/5	94.4 (1.1) 26/5	97.44 (0.43) 26/5	**100** **(0) 26/5**
		7	91.08 (1.18) 36/5	93.47 (0.88) 36/5	97.68 (0.45) 36/5	**100** **(0) 36/5**
		13	93.32 (1.16) 66/5	93.2 (1.47) 66/5	96.92 (0.5) 66/5	**100** **(0) 66/5**

		Total

		Max	97.72	97.73	99.28	100
		Min	91.08	91.47	96.24	98.7
		Average	94.73	94.81	97.94	99.92
		SD	1.78	1.63	0.75	0.26

**Table 8:** A comparison of the results obtained by our method and others' methods (classification accuracy) for two and three categories classification problems.

Classes	Authors (year)	Method	Dataset	Accuracy
**2**	Nigam et al. [15](2004)	Nonlinear preprocessing filter, diagnostic artificial neural network (LAMSTAR)	Z, S	97.2
	Srinivasan et al. [14] (2005)	Time & frequency domain features, recurrent neuralnetwork (RNN)	Z, S	99.6
	Kannathal et al. [42] (2005)	Entropy measures, adaptive neurofuzzy inference system (ANFIS)	Z, S	92.22
	Kannathal et al. [35] (2005)	Chaotic measures, surrogate data analysis	Z, S	~90
	Polat et al. [16](2006)	Fast Fouriertransform (FFT), decision tree(DT)	Z, S	98.72
	Subasi [22](2007)	Discrete wavelettransform (DWT), mixture ofexpert model	Z, S	95
	**This work (2007)**	**Time frequency (TF) analysis, artificial neuralnetwork (ANN)**	**Z,S**	**100**
	**This work (2007** **)**	**Time frequency (TF) analysis, artificial neuralnetwork (ANN)**	**(Z,O, N, F), S**	**97.73**

**3**	Guler et al. [34](2005)	Lyapunov exponents, recurrent neural network(RNN)	Z, F, S	96.79
	Sadati et al. [23](2006)	Discrete wavelettransform (DWT), adaptive neuralfuzzy network (ANFN)	Z, F, S	85.9
	**This work (2007)**	**Time frequency (TF) analysis, artificial neuralnetwork (ANN)**	**Z,F, S**	**99.28**
	**Thiswork (2007** **)**	**Time frequency (TF) analysis, artificial neuralnetwork (ANN)**	**(Z,O),** **(N,F), S**	**97.72**
